# Unusual Metastatic Sites of Testis and Rectum in Prostate Cancer Detected by ^68^ Ga-PSMA-11 PET/CT Imaging at Initial Staging

**DOI:** 10.1055/s-0044-1778710

**Published:** 2024-01-22

**Authors:** Rahul V. Parghane, Sandip Basu

**Affiliations:** 1 Radiation Medicine Centre, Bhabha Atomic Research Centre, Tata Memorial Hospital Annexe, Parel, Mumbai, Maharashtra, India; 2 Homi Bhabha National Institute, Mumbai, Maharashtra, India

**Keywords:** prostate cancer, prostate-specific membrane antigen, ^68^ Ga-PSMA-11 PET/CT, theranostics, metastatic disease to the testes and rectum

## Abstract

Imaging plays a pivotal role in defining the extent of disease and deciding therapeutic strategies in recently diagnosed high-risk prostate cancer. Standard-of-care conventional imaging may often miss rare metastatic disease sites. We herein present a unique case of prostate cancer where ^68^ Ga-PSMA-11 positron emission tomography (PET)/computed tomography (CT) detected two unusual metastatic sites (testis and rectum) in a single patient at initial staging, resulting in an accurate determination of the extent of disease, more tailored multimodal treatment planning, and exploration of the theragnostic potential.

## Introduction

In prostate cancer, the role of imaging is important for defining the extent of disease spread and subsequently deciding therapeutic strategies in recently diagnosed high-risk cases. One problem in the management of prostate cancer is a high relapse rate following curative-intent surgery or radiotherapy treatment. 1 2 This may be mainly related to the insufficient sensitivity and specificity of standard-of-care conventional imaging (computed tomography [CT] and bone scan) for detecting nonlocalized disease 3 and may be because of unconventional metastatic spread to rare sites that were missed on conventional imaging. We present here a rare case of prostate cancer with unusual metastatic sites (testis and rectum) detected by ^68^ Ga prostate-specific membrane antigen 11 ( ^68^ Ga-PSMA-11) positron emission tomography (PET)/CT imaging at initial staging.

## Case Report

**Fig. 1:**
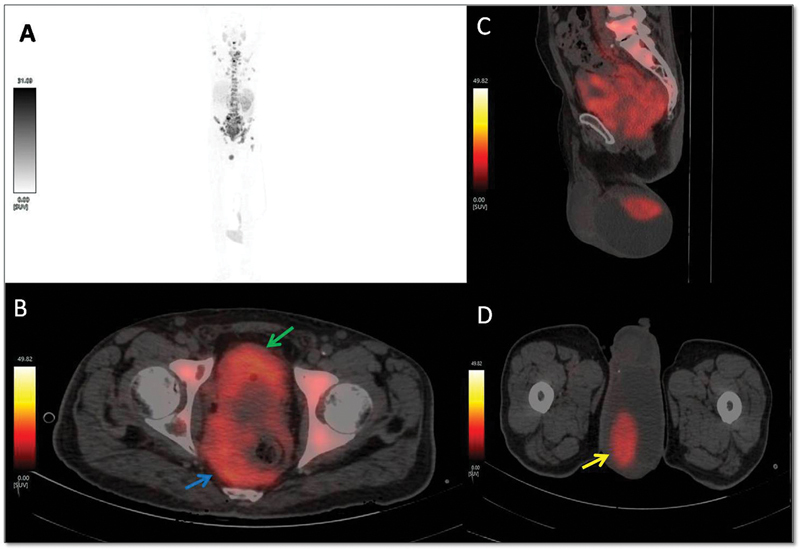
( **A** ) Maximum-intensity projection image of ^68^ Ga-PSMA-11 positron emission tomography (PET)/computed tomography (CT) scan showing extensive prostate-specific membrane antigen (PSMA) uptake in skeleton, abdominopelvic, and scrotal regions. ( **B** ) Transaxial image of ^68^ Ga-PSMA-11 PET/CT showing PSMA-avid (SUVmax-19) large-sized prostatic mass lesion ( *green arrow* ) and rectal lesion ( *blue arrow* ). Scrotal PSMA uptake (SUVmax-18) localized to the left testicular lesion ( *yellow arrow* ) with associated hydrocele shown in fused ( **C** ) sagittal and ( **D** ) transaxial PET/CT.

A 66-year-old man presented with lower urinary tract symptoms and lower limb swelling of more than 6 months' duration. Pelvic ultrasonography showed an enlarged prostate gland and there was raised serum prostate specific antigen (PSA) level of 217 ng/mL. Biopsy from the prostate gland lesion diagnosed prostatic adenocarcinoma with Gleason score of 4 + 5 = 9. The patient was referred for ^68^ Ga-PSMA-11 PET/CT scan for initial staging. Maximum-intensity projection image ( Fig. 1A ) and fused transaxial ( Fig. 1B ) image of ^68^ Ga-PSMA-11 PET/CT scan showed PSMA-avid large-sized prostatic mass lesion and rectal lesion. Fused sagittal ( Fig. 1C ) and transaxial ( Fig. 1D ) PET/CT images showed focal intense PSMA-avid left testicular lesion with associated hydrocele. PSMA-avid extensive metastatic skeletal lesions and abdominopelvic lymph nodes were seen in maximum-intensity projection ( Fig. 1A ) and fused sagittal ( Fig. 1C ) images of ^68^ Ga-PSMA-11 PET/CT scan.

## Discussion

In prostate cancer, metastatic spread to the testes and the rectum are rarely reported, which is ascribed to the lower temperature of the scrotum, the presence of blood–testis barrier formed by the Sertoli cells, 4 5 and critical barrier of the rectoprostatic (Denonvilliers') fascia between the prostate and the anterior rectal wall, 6 7 and involvement of the testes or rectum usually indicates advanced prostatic disease. PSMA is a cell surface glycoprotein normally present at a low level of expression on the prostate gland, but it is overexpressed on primary as well as metastatic lesions in prostate cancer. PSMA-targeting PET-based radioligands show higher affinity for binding to overexpressed PSMA in prostate cancer, forming the basis for whole-body tumor imaging with a higher tumor-to-background contrast, higher specificity, and the ability to identify low-volume visceral and bone metastatic disease. These features result in highly sensitive and specific PET/CT imaging for detecting primary as well as metastatic lesions in prostate cancer patients. 8 9 10

Nevertheless, recent findings suggest that PSMA PET may not exhibit the anticipated level of specificity for detecting prostate cancer. There has been growing evidence of nonprostatic disorders demonstrating PSMA expression. These disorders include inflammatory/infectious diseases, vascular conditions, benign neoplasms, and various malignancies. Particularly related to our case are inflammatory/infectious disease of the rectum and scrotum, primary rectal carcinoma, and testicular tumors, etc. 11 Therefore, a histological examination is crucial for the ultimate diagnosis of PSMA-avid lesions, especially in cases of oligometastatic disease where it may alter the course of therapy. As mentioned in the literature, PSMA uptake by second primary tumors is frequently lower than that by prostate cancer metastases. 10 11 In our case, there was extensive metastatic disease involving a widely disseminated skeletal system. Additionally, the intensity of PSMA expression in prostatic lesions and skeletal lesions was like the intensity of PSMA uptake in rectal and scrotal lesions, suggesting prostate metastatic disease.

## Conclusion

^68^ Ga-PSMA-11 PET/CT imaging is a highly effective imaging modality for detecting usual as well as unusual metastatic sites and defining the extent of disease. This aids in more tailored multimodal treatment planning for prostate cancer patients, including PSMA-based theranostics.
